# Effect of *Flammulina velutipes* Soluble Dietary Fiber on Dough Processing Characteristics and Micro-Fermented Dried Noodles Quality Properties

**DOI:** 10.3390/foods13172764

**Published:** 2024-08-30

**Authors:** Wenliang Wang, Shuang Yang, Lihong Wang, Furong Hou, Shasha Song, Yansheng Wang, Zhiqing Gong, Fengjuan Jia

**Affiliations:** 1 Institute of Agro-Food Science and Technology, Shandong Academy of Agricultural Sciences, Jinan 250100, China; cywwl@163.com (W.W.);; 2 Department of Life Science and Food Engineering, Hebei University of Engineering, Handan 056200, China

**Keywords:** soluble dietary fiber, micro-fermented dried noodles, farinograph properties, dynamic rheology properties, nutrition, flavor

## Abstract

Our research focused on the integration of *Flammulina velutipes* soluble dietary fiber (Fv-SDF) into wheat flour during the production of dried noodles, delving into the impact of different addition ratios of Fv-SDF on both dough processing characteristics and the quality of the micro-fermented dried noodles. The viscometric and thermodynamic analyses revealed that Fv-SDF notably improved the thermal stability of the mix powder, reduced viscosity, and delayed starch aging. Additionally, Fv-SDF elevated the gelatinization temperature and enthalpy value of the blend. Farinograph Properties and dynamic rheology properties further indicated that Fv-SDF improved dough formation time, stability time, powder quality index, and viscoelasticity. Notably, at a 10% Fv-SDF addition, the noodles achieved the highest sensory score (92) and water absorption rate (148%), while maintaining a lower dry matter loss rate (5.2%) and optimal cooking time (142 s). Gas chromatography-ion mobility spectrometry (GC-IMS) analysis showed that 67 volatile substances were detected, and the contents of furfural, 1-hydroxy-2-acetone, propionic acid, and 3-methylbutyraldehyde were higher in the Fv-SDF 10% group. These 10% Fv-SDF micro-fermented noodles were not only nutritionally enhanced, but also had a unique flavor. This study provides a valuable theoretical basis for the industrial application of *F. velutipes* and the development of high-quality dried noodles rich in Fv-SDF.

## 1. Introduction

*Flammulina velutipes* is known as enoki mushroom, golden mushroom, belonging to Basidiomycotina, Agaricales, Tricholomataceae, and Flammulina [1]. It is cultivated at large scales in East Asia, especially China, Japan, Vietnam and Korea [2]. *F. velutipes* is also one of the major edible mushrooms employed in factory cultivation, and it has medicinal, edible, and ornamental value, with immeasurable commercial potential [3]. *F. velutipes* contains many healthy nutrients, including dietary fiber, vitamins, minerals, organic acids, and other biologically active components [4]. The contents of polysaccharides and peptides in *F. velutipes* are 7% and 15%, respectively [5]. There is a wide range of biological as well as pharmacological activities associated with these compounds, such as antifungal, anticancer, anti-tumor, anti-inflammatory, and antioxidant properties [6]. Dietary fiber (DF) is one of the main nutritional components of *F. velutipes*, accounting for 32% of dry *F. velutipes* body [7]. Compared with the polysaccharides and peptides in *F. velutipes*, the rich content of DF is rarely mentioned and has not been effectively utilized.

The term DF refers to indigestible carbohydrates and lignin, which could be categorized as plant dietary fiber, animal dietary fiber, algae dietary fiber, or microbial dietary fiber [8]. According to the amount of water they can saturate, DF is commonly classified as insoluble (IDF) or soluble (SDF) [9]. IDF is principally composed of cellulose, lignin, and insoluble hemicellulose, while SDF is composed of soluble hemicellulose, pectin, gum, and oligosaccharides [10]. DF is a nonnutritive component of food, and several health benefits have been associated with DF. Studies have shown that DF can effectively inhibit the absorption of cholesterol in the digestive tract, prevent it from entering the bloodstream, and effectively prevent cardiovascular and cerebrovascular diseases caused by arteriosclerosis [11]. DF has good water and fat solubility and can combine with sugars and oils in the intestine to reduce the absorption of sugars and oils in the intestine [12]. It has been suggested that DF consumption could contribute to diabetes prevention by increasing satiety, reducing nutrient absorption, and decreasing weight [13]. In addition, DF also has a certain role in reducing the incidence of colon cancer, and breast cancer [14]. On the other hand, SDF has a broad application prospect as a food additive, stabilizer, gelling agent, and thickener in food processing based on its advantages of water retention, oil retention, and structural characteristics [10]. Moreover, the addition of SDF can extend the shelf life of fat-rich foods by increasing the antioxidant capacity of the emulsion [15]. The SDF of *F. velutipes* (Fv-SDF) is mainly composed of glucose, hemicellulose, dextran, pectin, oligosaccharides, etc, and the application of Fv-SDF in food processing has been rarely studied.

In the past few decades, people have been blindly pursuing fine grains, and many nutrients of traditional wheat flour have been seriously lost under various fine processing, especially dietary fiber [16]. The loss of nutrients is directly related to the eating quality and nutrition of flour products. SDF has high gelation, can be used as an emulsifier, and is easy to combine with food systems [17]. SDF is filled between the protein matrix and starch granules in the form of a binder. Although DF content in wheat flour is only 10–15%, it greatly influences the quality and taste of flour products [18]. Dried noodles are the staple food of many Asian countries and are one of the most produced and consumed flour products in China [19]. Considering the deep processing of grain and the sub-health of people, the importance of DF in grain processing and diet is highlighted [20]. At present, most of the research focuses on the influence of grain DF on the quality characteristics of ordinary dried noodles, while research on the influence of fruit and vegetable DF on the quality of micro-fermented dried noodles is rare. In recent years, micro-fermented dried noodles are one of the most popular high-quality dried noodles in the market, with a smooth appearance without depressions, and many fine holes in the internal structure [21]. The main advantages of micro-fermented dried noodles are enhanced nutritional value, improved digestion and absorption, rich taste, and easy digestion. Micro-fermentation technology, with its unique production technology, not only retains the advantages of traditional noodles but also significantly improves the flavor and texture of fermentation. Micro-fermented dried noodles have great development potential in the dried noodles market because of their good taste and suitability for both young and old people. However, no studies have been done to improve the quality of dried noodles by both adding Fv-SDF and applying micro-fermentation technology.

In this study, the effects of different doses of Fv-SDF on dough processing characteristics and the cooking characteristics of micro-fermented dried noodles were investigated. The application of Fv-SDF in food processing extended the edible mushroom industry chain and promoted the development of the edible mushroom industry, which was of great significance for the development of the edible mushroom processing industry. Fv-SDF micro-fermented dried noodles not only satisfied consumers’ demand for nutritionally fortified foods but also enriched the varieties of dried noodles, which laid a foundation for developing dried noodles with more nutritional and health care functions and provided a reference for the application of edible fungi in food.

## 2. Materials and Methods

### 2.1. Preparation of Mixed Flour Dough and Fv-SDF Micro-Fermented Dried Noodles

The Fv-SDF was extracted from *F. valutipes* by ultrasound-assisted subcritical water extraction following the method of Yan et al. [22]. The noodles of Ginindza et al. [23] were adopted and improved. The mixed system of Fv-SDF and wheat flour (Jinlongyu Grains and Oils Food Co., Shanghai, China) was prepared by adding Fv-SDF to wheat flour at the proportions of 0%, 5.0%, 7.5%, 10.0%, 12.5%, and 15.0%, and mixing them evenly. Then, 1.0% salt, 1.2% yeast, and 35 mL distilled water were added in proportion. The mixture was mixed evenly, placed into a bread maker (EGBM010 bread machine, Electrolux Electric Co., Ltd., Shanghai, China), and stirred for 10 min. After the dough was mixed, it was kneaded by hand, and then put in the fermentation chamber (6D Fermentation chamber, Deer Ma Appliances, Foshan, China) for 9 min. The noodles were made with a noodle maker. The noodles were dried in a 40 °C blast drying oven (GZX-9240MBE Electric Blast Drying Oven, Shanghai, China) for 4 h to prepare Fv-SDF micro-fermented dried noodles.

### 2.2. Differential Scanning Calorimeter Analysis

DSC (DSC-250, TA Instruments-Waters, New Castle, NC, USA) was used to determine the thermodynamic properties of the mix powder [24]. The solution of the 4.0 mg sample (mix powder:water = 5 g:10 mL) was placed in a crucible and stored overnight at 4 °C to make the sample fully expand. During the measurement, the sample was heated from 25 °C to 100 °C at the speed of 10 °C/min. The samples were measured three times to obtain thermodynamic parameters. The thermodynamic parameters included initial gelatinization temperature (T_0_/°C), peak temperature (Tp/°C), terminating gelatinization temperature (Tc/°C), and gelatinization enthalpy (ΔH, W/g).

### 2.3. Rapid Analysis of Viscosity

The gelatinization properties of the mixture were measured by RVA [25]. Then, 3 g of Fv-SDF complex and wheat flour were weighed and 25 mL of distilled water was added to make the total weight 28 g. Then, it was put into a rapid viscosity analyzer (RVA-Tec Master, Perten, Australia) for testing. The gelatinization properties included peak viscosity (cp) trough viscosity (cp), breakdown value (cp), final viscosity (cp), setback value (cp) peak time (min), and pasting temperature (°C).

### 2.4. Determination of Farinograph Properties

Farinograph (Micro-dough LAB, Sweden porton corp, Stockholm, Sweden) was used to measure the farinograph properties of the mixture, following the method of Zhang et al. [26] with slight modifications. The mixed system of Fv-SDF wheat flour was prepared by adding Fv-SDF to wheat flour at the ratios of 0%, 5.0%, 7.5%, 10.0%, 12.5%, and 15.0%. An amount of 50 g of the powder mixture was placed in the farinograph and the rotation speed was set at 61~65 r/min. The farinograph properties included consistency (FU), water absorption (%), dough development time (min), stability time (min), weakening degree (FU), and flour quality index (mm).

### 2.5. Determination of the Dynamic Rheology Properties

The dynamic rheology properties were measured according to Huang et al. [12,27] with slight modifications. Then, 4 g of Fv-SDF micro-fermented dough (mix powder:water = 100:40) was put in a rheometer (HR 20 Rheometer, TA Instruments, Waters Corporation, Milford, MA, USA) to determine its storage modulus (G′), loss modulus (G″) and loss tangent (tanδ). The frequency scanning was 0.1~500%, and the testing temperature was 25 °C.

### 2.6. Apparent Color Detection of Dried Noodles

The effect of the addition of Fv-SDF on the chromaticity value of micro-fermented dried noodles was measured by a portable precision colorimeter (CR-400, Konica Minolta, Tokyo, Japan) [28]. The CIE-Lab color space represents the color characteristics of noodles, with the L* value representing whiteness, the a* value representing red-greenness, and the b* value representing yellow-blue intensity. The probe of the colorimeter measured the samples of micro-fermented noodles and recorded the values of L*, a*, and b*. Each sample was tested 3 times, and the average value of the 3 tests was the final result.

### 2.7. Determination of the Best Cooking Time for Dried Noodles

The method of Noonim et al. [29] was used and improved. 10 g noodles were boiled in 500 mL of water for 1 min, and then a noodle was taken out every 15 s. The best cooking time for noodles is when the white starch core of the noodles disappears.

### 2.8. Determination of the Cooking Characteristics of Dried Noodles

The cooking characteristics of micro-fermented dried noodles were determined according to the method of Wang et al. [30] with slight modifications. 20 g of Fv-SDF micro-fermented dried noodles were put into 1 L of boiling water and cooked at the best cooking time. The water absorption and cooking loss rate of the noodles were calculated according to the following Formulas (1) and (2), respectively.

(1) $$\mathrm{Water}\;\mathrm{absorption}=\frac{m_{2}-m_{1}}{m_{1}}\times 100\%$$

(2) $$\mathrm{Cooking}\;\mathrm{loss}=\frac{m_{1}-m_{3}}{m_{1}}\times 100\%$$

where *m*_1_ is the dry weight/g of Fv-SDF micro-fermented dried noodles; *m*_2_ is the cooked mass/g of Fv-SDF micro-fermented dried noodles; *m*_3_ is the dry weight/g of Fv-SDF micro-fermented dried noodles after cooking.

### 2.9. Determination of Sensory Evaluation of Dried Noodles

According to the method proposed by Yeoh et al. [31], the sensory evaluation of Fv-SDF micro-fermented dried noodles was determined. Six men and six women were selected for sensory evaluation of the noodles, and the scoring criteria are shown in Table S1. Twelve healthy panelists (6 females and 6 males, 20–28 years of age) were recruited from the Institute of Agro-Food Sciences and Technology, Shandong Academy of Agricultural Sciences (Jinan, China). Before the sensory test, these 12 members needed to undergo a week-long systematic sensory evaluation training according to the GB/T 16291.1-2012 standard [32]. All sensory tests were conducted in an air-conditioned room (22 °C) with separate compartments. We adhered to the ethical principles of sensory research at the Agro-Food Sciences and Technology, Shandong Academy of Agricultural Sciences. These principles were reviewed by the Research Ethics Committee at the Institute of Agro-Food Sciences and Technology, Shandong Academy of Agricultural Sciences (Statement 11/2023). Before participating in this study, each participant provided written informed consent.

### 2.10. Determination of Flavor Compounds of Dried Noodles

According to the descriptive analysis method of Hou et al. [32], the flavor substances of Fv-SDF micro-fermented dried noodles were determined. The volatile compounds were analyzed by chromatography ion mobility spectrometry (GC-IMS) instrument (Gesellschaftfür Analytische Sensorsysteme mbH (G.A.S.), Dortmund, Germany). An amount of 3 g sample was put in a 20 mL headspace bottle, incubated at 80 °C for 15 min, and then driven by N_2_ into the chromatographic column for detection. The NIST (2020) and IMS databases (0.4.07) in the instrument software were used for a qualitative analysis of volatile compounds [33].

### 2.11. Data Analysis

The test results were repeated at least 3 times, and the final results were shown as mean ± standard deviation (SD). SPSS 24.0 (SPSS Inc., Chicago, IL, USA) was used for one-way ANOVA, the Duncan method was used for differences between groups, and *p* < 0.05 showed significant differences.

## 3. Results and Discussion

### 3.1. Effect of Fv-SDF Addition on Thermodynamic Properties of Wheat Starch

**Figure 1:**
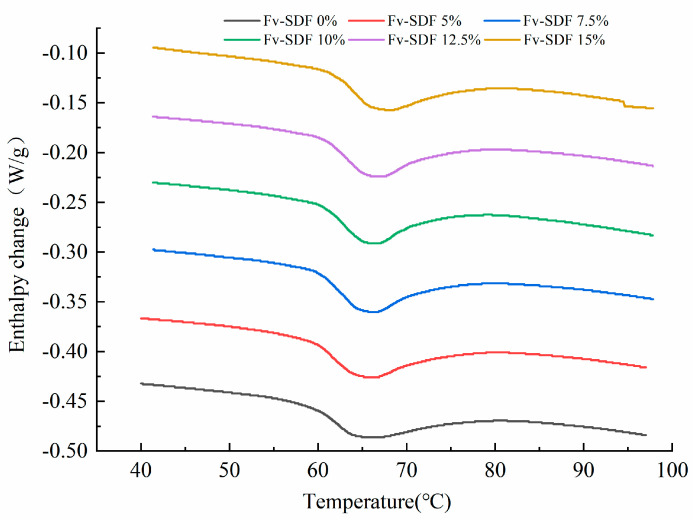
Effect of Fv-SDF addition on the thermodynamic properties of wheat starch.

In order to better understand the effect of Fv-SDF on the quality of dried noodles, the thermodynamic properties of Fv-SDF on wheat starch were first studied. The thermodynamic properties can reflect the enthalpy change and moisture form of the mixed dough during crystallization and melting [34]. The peak temperature is the temperature at which the sample absorbs heat during gelatinization, the gelatinization enthalpy (ΔH) reflects the aggregation degree of protein and also shows the hydrophobic and hydrophilic properties of protein [35]. The results showed that the peak temperature, gelatinization temperature, and gelatinization enthalpy of the mix powder increased with the Fv-SDF addition ratio (Table 1 and Figure 1), but gelatinization enthalpy did not increase significantly. The initial gelatinization temperature T_0_, peak temperature Tp, terminating gelatinization temperature Tc, and gelatinization enthalpy ∆H of the mix powder were 56.32 °C, 63.65 °C, 69.40 °C, and 1.80 W/g, respectively. With the increase of FV-SDF addition to 15%, the T_0_, Tp, Tc, and ∆H of the mix powder increased to 61.55 °C, 67.12 °C, 73.26 °C, and 2.22 W/g, respectively. The increase in gelatinization temperature was consistent with the RVA results, indicating that a higher gelatinization temperature was required during the gelatinization process of wheat flour. This was due to the fact that Fv-SDF competed with proteins for water absorption, which led to the decrease of available water in proteins, thus increasing the energy needed for protein denaturation and increasing the gelatinization temperature of the mixture. These results were consistent with the findings of Wang et al. [36], which studied the effect of soluble soybean polysaccharides on starch gelatinization and found that the gelatinization temperature of the starch system increased, indicating that the addition of polysaccharides increased the difficulty of starch gelatinization.

### 3.2. Effect of Fv-SDF Addition on Pasting Properties of Wheat Starch

**Figure 2:**
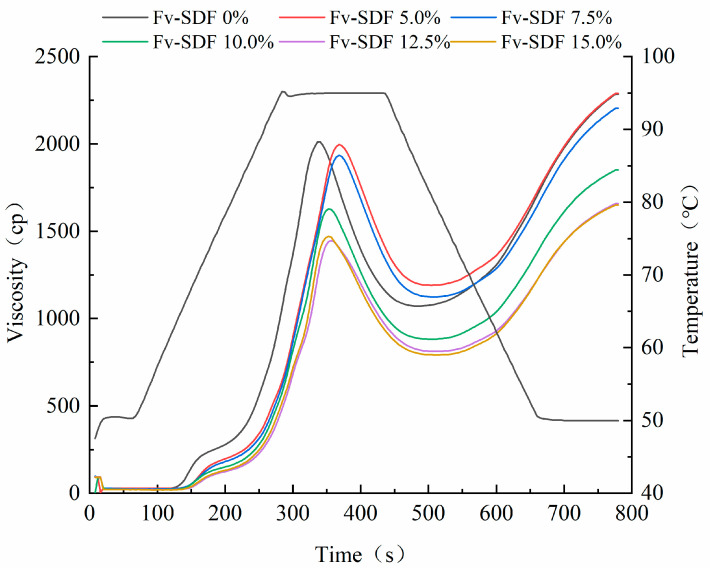
Effect of Fv-SDF addition on the pasting properties of wheat starch.

Secondly, the influence of Fv-SDF on the pasting properties of wheat starch was detected. The pasting properties mainly reflect the swelling ability of starch in the composite flour system and the binding ability of starch with water. In addition, it can also reflect the effect of starch gelatinization characteristics on flour products [37]. As shown in Figure 2, the viscosity of starch paste gradually increased with the extension of heating time; the viscosity peak appeared at 300–400 s and moved down with the increase of Fv-SDF addition. With the further extension of heating time, the viscosity of the starch paste decreased and the viscosity trough appeared at 450–600 s. With the increase of Fv-SDF addition, the viscosity trough moved down. When the heating time was 450–600 s, the fluidity of the starch paste decreased, while the viscosity increased.

The essence of starch gelatinization is that water enters the crystallization region of starch particles and the internal structure of starch changes from ordered to disordered [38]. As shown in Table 2, with the increase of Fv-SDF content, the viscosity and setback value of the mix powder showed a downward trend. The decrease in viscosity may be due to the fact that the addition of Fv-SDF diluted the starch content of wheat and reduced the cross-linking degree of the starch structure in the mixed paste, resulting in a decrease in viscosity of the mixture [39]. In addition, the amylose content also contributed to the reduction of viscosity, which was consistent with other research results [40]. The setback value is an important indicator of short-term retrogradation/aging of amylose [41]. Fv-SDFs, starches, and proteins interacted to form new macromolecules, which hindered the interaction between starch molecules and delayed the formation of crystals. The breakdown value reflects the stability of the starch paste, and Fv-SDF improved the thermal stability of the mixture. Our results were consistent with the findings of Wang et al. [42], that is, adding polysaccharide to flour can reduce viscosity.

### 3.3. Effect of Fv-SDF Addition on Farinograph Properties of Mix Powder

The farinograph properties of wheat flour reflect the changes in rheological characteristics during dough mixing and are one of the important indexes to evaluate the processing characteristics of wheat flour [43]. The farinograph properties of the mixture of Fv-SDF and wheat flour are shown in Table 3. With the increase of Fv-SDF addition, the dough stability time, dough development time, and flour quality index increased, while the water absorption and weakening degree decreased. The stability time of dough is closely related to the toughness and gluten strength of the dough [44]. Our results showed that the addition of Fv-SDF could prolong the stability time of dough, improve the mechanical stirring resistance of dough, and enhance gluten strength. In addition, Fv-SDF is a kind of gel polysaccharide, which can connect the gluten network structures, delay the breaking of disulfide bonds in the dough, and the depolymerization of gluten macromolecules, making the gluten network more stable [45]. The dough development time refers to the time required for stirring from the beginning of adding water to the maximum consistency of dough [46]. With the increase of the dosage of Fv-SDF, the dough development time tended to increase, which was because the high water-holding capacity of Fv-SDF reduced the water absorption of the gluten protein, prolonged the dough formation time, and further affected the formation rate of the gluten network. These results were similar to those obtained by adding bean dregs, apple dregs, and oats to wheat flour [47]. The decrease in water absorption of dough caused by Fv-SDF was mainly due to the fact that Fv-SDF was more hydrophilic than starch. Fv-SDF could form a barrier layer around the starch granules, which prevented water molecules from contacting the starch granules and limited the expansion of the starch granules. In addition, previous studies also reported that adding a high content of sugar and fiber to wheat flour could increase the stability time of dough and decrease water absorption [48]. To sum up, adding Fv-SDF to wheat flour was beneficial to the formation of a gluten network, which could not only improve gluten strength but also enhance the nutritional characteristics of the mixed flour. Therefore, Fv-SDF could be used as an improver for medium and low gluten flours.

### 3.4. Effect of Fv-SDF Addition on Dynamic Rheological Properties of Dough

**Figure 3:**
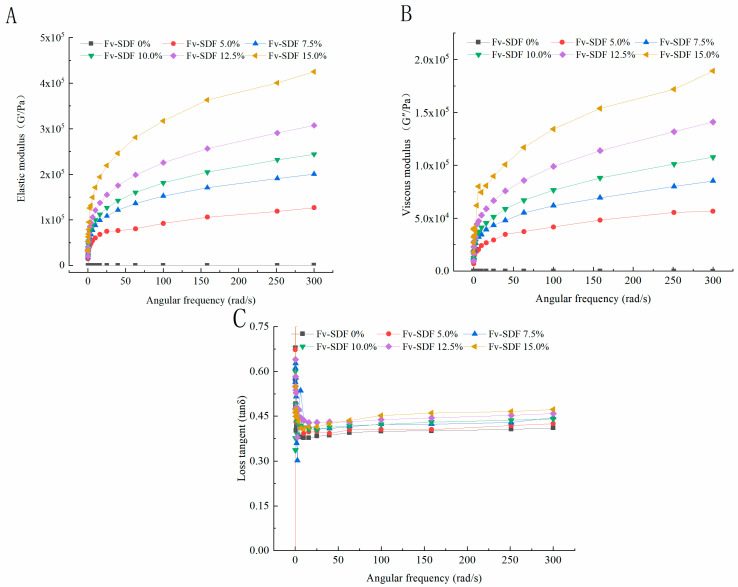
Effects of Fv-SDF addition on the storage modulus (G′) (**A**), loss modulus (G″) (**B**), and loss tangent (tanδ = G″/G′) (**C**) of dough.

The dynamic rheological properties of dough mainly refer to its viscoelasticity. The viscoelasticity of dough could characterize its structure and physicochemical properties, that is, the tight combination of moisture and gluten, thus predicting the changes in dough processing and the quality of products [49]. As shown in Figure 3, with the increase of angular frequency, the storage modulus (G′) (Figure 3A) and the loss modulus (G″) (Figure 3B) of the samples showed an upward trend. The increase in dynamic modulus was due to the fact that the hydroxyl groups in Fv-SDF changed the moisture distribution of the dough. Besides, Fv-SDF crosslinked with the protein in wheat flour to form a relatively stable gel structure, which enhanced the elasticity of gluten protein and led to the increase of G′ and G″ [50]. In addition, the G′ of the same sample was obviously higher than that of G″, and the overall performance was elasticity [51]. When the addition of Fv-SDF was 10%, the viscoelasticity of the dough was better and the quality of the dried noodles was excellent. As shown in Figure 3C, with the increase of Fv-SDF addition, the values of loss tangent (tanδ = G″/G′) showed an upward trend. However, the tanδ values were all less than 1, indicating that the elasticity of the dough was dominant. Those results indicated that the Fv-SDF mixed dough system was more stable and the micro-fermented dried noodles were not easy to break in the low-frequency scanning range. To sum up, the addition of Fv-SDF can increase the ductility of the dough.

### 3.5. Effect of Fv-SDF Addition on Apparent Color of Dried Noodles

The effect of Fv-SDF addition on the whiteness of micro-fermented dried noodles was determined by colorimeter. Where L* is the whiteness, with a value range of 0–100, and the greater the value, the whiter the color. a* is the red-green value, the negative value represents green, and the positive value represents red. b* is the yellow-blue value, the negative value indicates that the color is blue, and the positive value indicates that the color is yellow. As shown in Table 4, compared with the control group, with the increase of Fv-SDF addition, the whiteness value L* of Fv-SDF micro-fermented dried noodles decreased significantly, while a* and b* increased significantly. The L* value of Fv-SDF powder was the lowest, and the values of a* and b* were the highest. Among them, L*, a*, and b* were all positive values, indicating that the whiteness of Fv-SDF micro-fermented dried noodles was reduced and that it was reddish and yellowish. Compared with the high Fv-SDF addition, the effect of low Fv-SDF addition on the apparent color of micro-fermented dried noodles was more obvious. In summary, when the addition of FV-SDF was 10%, the appearance of micro-fermented dried noodles was more pleasant.

### 3.6. Effect of Fv-SDF Addition on the Cooking Quality of Dried Noodles

**Figure 4:**
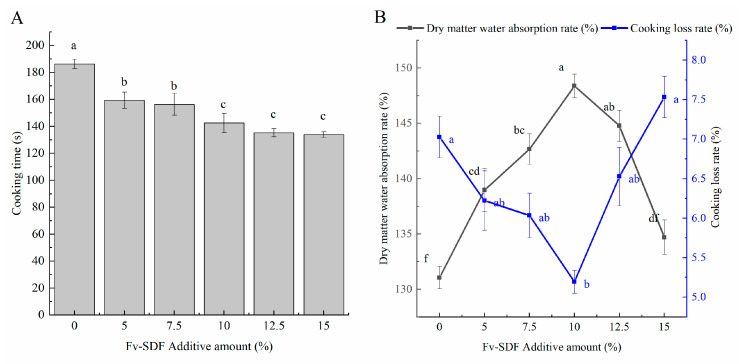
The effect of Fv-SDF addition on the optimum cooking time (**A**), water absorption, and cooking loss rate (**B**) of dried noodles. Different letters represent significance (*p* < 0.05).

The best optimum cooking time is the time when the white core of the noodles disappears completely during steaming [52]. As shown in Figure 4A, with the increase of the Fv-SDF addition, the optimum cooking time of Fv-SDF micro-fermented dried noodles gradually decreased. The addition of Fv-SDF diluted the starch content, and the decrease in starch content led to a decrease in gelatinization temperature. After micro-fermentation, there were many air holes in the noodles, and the water molecules could easily enter the inside, which made the starch absorb water and swell rapidly, reducing the optimal cooking time [53]. When the addition of Fv-SDF was 10%, the optimum cooking time of dried noodles was 142 s. The addition of Fv-SDF had a significant effect on the water absorption and cooking loss rate of micro-fermented dried noodles. Our results showed that when the addition of Fv-SDF was 10%, the dry matter water absorption of micro-fermented dried noodles was the highest and the cooking loss rate of micro-fermented dried noodles remained at a low level (Figure 4B). With the further increase of Fv-SDF addition, the water absorption of micro-fermented dried noodles showed a downward trend, while the cooking loss rate showed an upward trend. FV-SDF had high water absorption, which was able to absorb water and form a gelatinous substance, and lock the moisture in the noodles, making the noodles absorb water and mature faster. The addition of Fv-SDF could form a protective net around the starch particles, thus reducing the spontaneous rupture of expanded particles, reducing the dissolution rate of starch, and reducing the loss rate of micro-fermented dried noodles during the cooking.

**Figure 5:**
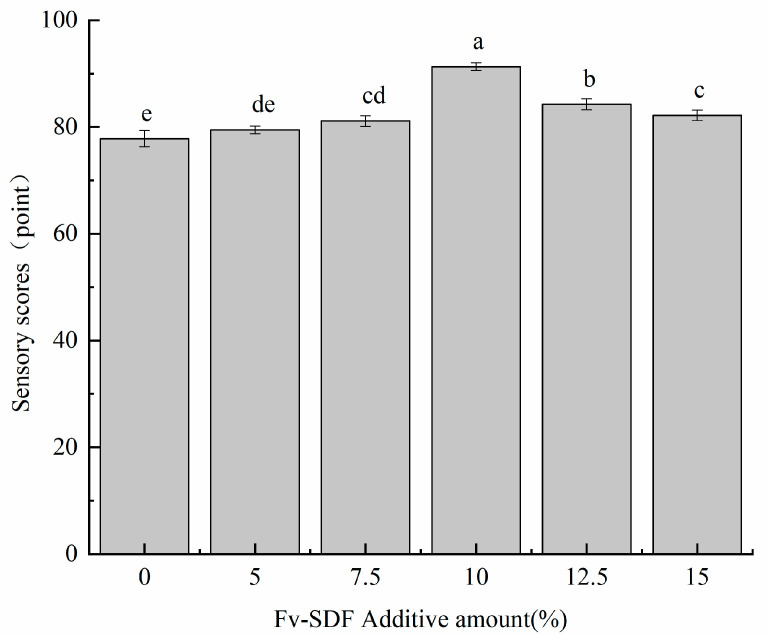
Effects of Fv-SDF addition on sensory evaluation of dried noodles. Different letters represent significance (*p* < 0.05).

The results of the sensory evaluation are shown in Figure 5. With the increase of the addition of Fv-SDF, the sensory score of Fv-SDF micro-fermented dried noodles generally showed a trend of increasing first and then reducing. When the addition of Fv-SDF was less than 10%, the toughness and taste scores were higher. The addition of Fv-SDF enhanced the gluten strength, chewiness, and elasticity of Fv-SDF micro-fermented dried noodles, and at the same time made the noodles have mushroom flavor and micro-fermentation aroma. In addition, the appearance of Fv-SDF micro-fermented dried noodles was uniform, and the color gradually darkened, giving it an attractive caramel color. The sensory score of micro-fermented dried noodles decreased with the further increase of Fv-SDF addition, because excessive addition of Fv-SDF reduced the hardness, chewiness, and toughness, and the color of micro-fermented dried noodles was dark brown, resulting in a decrease in the sensory evaluation of Fv-SDF micro-fermented dried noodles. When the addition of Fv-SDF was 10%, the chewiness, elasticity, flavor, and color of the micro-fermented dry noodles were better, and the sensory scores of the micro-fermented dry noodles were the highest.

### 3.7. Comparative Analysis of Volatile Flavor Compounds in Dried Noodles

In order to further clarify the differences in volatile flavor compounds in dried noodles, the GC-IMS was selected to identify the characteristic peak areas of the samples. The three dried noodle samples were as follows: CK group (unfermented, Fv-SDF addition amount 0%), Fv-SDF 0% (micro-fermentation for 9 min, Fv-SDF addition amount 0%), and Fv-SDF 10% (micro-fermentation for 9 min, Fv-SDF addition amount 10%).

**Figure 6:**
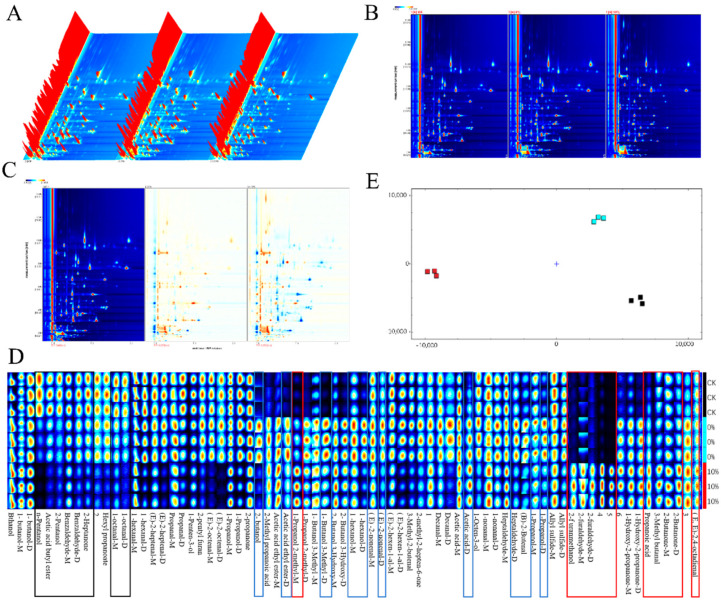
Three-dimensional spectrum of volatile substance composition (**A**), 2D spectrum of volatile substance composition (**B**), 2D differential spectrum of volatile substance composition (**C**), GC-IMS fingerprint (**D**), PCA analysis (**E**). In (**A**–**C**), the samples from left to right are the CK group, Fv-SDF 0% group, and Fv-SDF 10% group, respectively.

In the three-dimensional spectrum (Figure 6A) and two-dimensional spectral top view (Figure 6B,C) of volatile flavor compounds of dried noodles, obvious visual differences of volatile flavor compounds could be observed among different samples (CK, 0% and 10%) in the GC-IMS spectrum.

Figure 6D was a fingerprint of the volatile components in three samples of dried noodles, which represented the signal peak of each volatile substance in different samples. The darker the red color, the higher the concentration of the substance. By comparing and analyzing the volatile components in three samples of dried noodles, the results showed that there was no significant difference in the composition and content of flavor components between the CK group and the Fv-SDF 0% group, but there was a significant difference in the composition and content of flavor components between the CK group and Fv-SDF 10% group. It could be seen from Figure 6D that there was little difference in the content of ethanol and butanol in dried noodles. It could be seen from the red frame that compared with the CK group and Fv-SDF 0% group, the contents of furfural, 1-hydroxy-2-acetone, propionic acid, and 3-methylbutyraldehyde increased in the Fv-SDF 10% group. Among them, furfural, 1-hydroxy-2-acetone, and 3-methylbutyraldehyde provided caramel flavor and sweetness, and propionic acid provided yogurt and vinegar flavors. In addition, propionic acid may be the sour taste produced by the micro-fermentation, while furfural, 1-hydroxy -2-acetone, and 3-methylbutyraldehyde were produced by non-starch polysaccharides contained in Fv-SDF. In the blue frame, it could be seen that compared with the CK group and Fv-SDF 10% group, the contents of 2-butanol, 2-methylpropionic acid, ethyl acetate, 2-methylpropanol, 3-methylbutanol, 3-hydroxy-2-butanonend were higher in Fv-SDF 0% group. Among them, 2-methylpropionic acid provided a sour taste, ethyl acetate provided a fresh taste, and 2-methylpropionic acid provided a sour taste which was produced during the micro-fermentation. As can be seen from the black frame in Figure 6D, compared with the Fv-SDF 0% group and Fv-SDF 10% group, the contents of pentanal, butyl acetate, benzaldehyde, 2-heptanone, hexyl propionate, octanal, hexanal, and trans-2-heptenal were higher, which provided a clear fragrance, fruity flavor, and fatty taste. Compared with the CK group, the content of flavor substances in the Fv-SDF 0% group was higher, and the types were richer, which were mainly produced by micro-fermentation. Among them, 3-methylbutyraldehyde has a strong malt and yeast flavor, which is a product of protein hydrolysis and amino acid (isoleucine and leucine) degradation, and it is an important flavor compound in many fermentation products [54].

The PCA analysis of three samples was shown in Figure 6E, which is a dimensionality reduction method commonly used to reduce the dimension of large data sets. The results showed that the contribution rates of PC-1 and PC-2 were 63% and 34%, respectively, and the cumulative variance contribution rate was 97%, which indicated that the PCA results were reliable. The distribution of the PC-2 axis of the CK, Fv-SDF 0%, and Fv-SDF 10% group was significantly different. The CK and Fv-SDF 0% groups were close to each other on the PC-1 axis, and these two groups were farther away from the Fv-SDF 10% group on the PC-1 axis. There was a great difference in flavor between the CK and Fv-SDF 10% group, while there was a little difference between the Fv-SDF 0% and 10% group, which was consistent with the fingerprint analysis.

In order to further clarify the composition of flavor compounds in the three dried noodle samples, all flavor compounds were classified. As shown in Table 5, 67 volatile substances, including 20 alcohols, 21 aldehydes, 9 ketones, 4 esters, and 13 other compounds, were detected in all three samples. There were significant differences in the volatile components of the three dried noodle samples, but the flavor components in the CK and Fv-SDF 10% groups were more significant. Consistent with fingerprint analysis, the contents of furfural, 1-hydroxy-2-acetone, propionic acid, and 3-methylbutyraldehyde were higher in the Fv-SDF 10% group.

## 4. Conclusions

In this study, the effects of Fv-SDF on dough processing characteristics and micro-fermented dried noodles were investigated. The farinograph and rheological properties tests showed that the addition of Fv-SDF was positively correlated with dough stability time, development time, and the flour quality index, but negatively correlated with dough water absorption and weakening degree. When the addition of FV-SDF was 15%, the dough stability time was 6.7 min and the development time reached 5.6 min. Fv-SDF could improve the elasticity of dough and enhance the strength of gluten. RVA results showed that the retrogradation value of the mixed flour decreased with the increase of Fv-SDF content, which indicated that the addition of Fv-SDF had an anti-aging effect and prolonged the shelf life of the noodles. The addition of 10% Fv-SDF made noodles more acceptable with the highest sensory score of 92, including pleasant color and good cooking characteristics. When the addition of Fv-SDF was 10%, the optimal cooking time was 142 s, the water absorption rate of dried noodles reached 148%, and the loss rate reached 5.2%. The results of GC-IMS analysis showed that the unique source of flavor compounds of noodles was generated by Fv-SDF and micro-fermentation treatment. Adding 10% Fv-SDF not only improved the nutritional value of the noodles but also gave them a unique flavor. This research can provide technical support for the postharvest processing and product development of *Flammulina velutipes* and provide a technical reference and theoretical basis for the application of Fv-SDF in micro-fermented dried noodle products.

## Figures and Tables

**Table 1:** Effect of Fv-SDF addition on thermodynamic properties of wheat starch.

Fv-SDF Addition/%	Initial Gelatinization Temperature T_0_/°C	Peak Temperature Tp/°C	Terminating Gelatinization Temperature Tc/°C	Gelatinization Enthalpy/(ΔH, W/g)
0	56.32 ± 0.23 ^d^	63.65 ± 0.16 ^e^	69.40 ± 0.35 ^d^	1.80 ± 0.45 ^b^
5.0	59.58 ± 0.16 ^c^	65.38 ± 0.23 ^d^	71.60 ± 0.95 ^c^	2.14 ± 0.43 ^a^
7.5	59.89 ± 0.08 ^c^	65.89 ± 0.20 ^c^	72.02 ± 1.10 ^c^	2.29 ± 0.21 ^a^
10.0	60.81 ± 0.33 ^b^	66.56 ± 0.47 ^b^	73.47 ± 1.85 ^a^	2.30 ± 0.29 ^a^
12.5	61.06 ± 0.42 ^b^	66.66 ± 0.16 ^b^	72.17 ± 0.33 ^bc^	2.29 ± 0.14 ^a^
15.0	61.55 ± 0.79 ^a^	67.12 ± 0.23 ^a^	73.26 ± 0.72 ^ab^	2.22 ± 0.16 ^a^

Note: Different letters represent significance in the same column (*p* < 0.05).

**Table 2:** Effect of Fv-SDF addition on the pasting properties of wheat starch.

Fv-SDF Addition/%	Peak Viscosity/cp	Trough Viscosity/cp	Breakdown Value/cp	Final Viscosity/cp	Setback Value/cp	Peak Time/min	Pasting Temperature/°C
0	2015.0 ± 14.24 ^a^	1069.5 ± 23.54 ^a^	945.5 ± 7.78 ^a^	2250.0 ± 50.91 ^a^	1180.5 ± 47.38 ^a^	5.8 ± 0.14 ^a^	64.8 ± 1.87 ^e^
5.0	1962.5 ± 47.38 ^ab^	1168.5 ± 30.41 ^a^	809.0 ± 4.24 ^b^	2237.0 ± 74.95 ^a^	1106.0 ± 8.49 ^b^	6.1 ± 0.86 ^a^	69.3 ± 1.00 ^d^
7.5	1876.0 ± 82.02 ^b^	1031.5 ± 30.81 ^a^	844.5 ± 48.79 ^b^	1972.0 ± 38.10 ^ab^	940.5 ± 87.28 ^c^	5.9 ± 0.37 ^a^	76.9 ± 0.47 ^c^
10.0	1595.0 ± 46.67 ^c^	845.0 ± 52.33 ^b^	750.0 ± 5.66 ^c^	1706.0 ± 25.06 ^b^	861.0 ± 52.74 ^cd^	5.8 ± 0.10 ^a^	86.2 ± 1.34 ^b^
12.5	1481.0 ± 24.04 ^c^	850.0 ± 53.74 ^b^	631.0 ± 5.66 ^d^	1689.5 ± 42.43 ^b^	839.0 ± 11.31 ^d^	6.0 ± 0.10 ^a^	88.0 ± 1.23 ^a^
15.0	1488.0 ± 48.08 ^c^	799.5 ± 12.02 ^b^	688.5 ± 12.02 ^d^	1669.5 ± 27.58 ^b^	870.0 ± 15.56 ^cd^	5.9 ± 0.04 ^a^	87.6 ± 0.53 ^ab^

Note: Different letters represent significance in the same column (*p* < 0.05).

**Table 3:** Effect of Fv-SDF addition on the farinogram properties of mix powder.

Fv-SDF Addition/%	Consistency/ FU	Water Absorption (Adjust 500 FU/%)	Dough Development Time/min	Stability Time/min	Weakening Degree (Firs 10 min/FU)	Weakening Degree (ICC/after Reaching the Maximum 12 min)	Flour Quality Index/mm
0	491.3 ± 3.18 ^a^	62.2 ± 1.52 ^a^	3.2 ± 0.14 ^c^	2.8 ± 0.39 ^d^	93.5 ± 6.36 ^ab^	126.5 ± 7.78 ^a^	48.0 ± 1.41 ^e^
5.0	505.0 ± 5.66 ^a^	55.1 ± 1.38 ^b^	3.5 ± 0.25 ^bc^	3.3 ± 0.25 ^d^	109.3 ± 16.62 ^a^	137.5 ± 14.85 ^a^	52.3 ± 1.77 ^de^
7.5	507.8 ± 15.20 ^a^	51.8 ± 0.74 ^c^	3.7 ± 0.04 ^bc^	3.8 ± 0.42 ^cd^	92.0 ± 12.73 ^ab^	120.3 ± 16.62 ^ab^	57.0 ± 1.41 ^d^
10.0	497.5 ± 0.71 ^a^	49.2 ± 0.07 ^d^	4.5 ± 0.39 ^ab^	4.7 ± 0.53 ^bc^	78.8 ± 6.01 ^bc^	111.8 ± 7.42 ^abc^	66.5 ± 0.71 ^c^
12.5	490.0 ± 12.73 ^a^	46.6 ± 0.12 ^e^	4.9 ± 0.21 ^a^	5.5 ± 0.71 ^ab^	59.5 ± 7.78 ^cd^	97.3 ± 10.96 ^bc^	75.75 ± 5.30 ^b^
15.0	500.0 ± 7.07 ^a^	44.5 ± 0.85 ^e^	5.6 ± 0.88 ^a^	6.7 ± 0.57 ^a^	43.5 ± 4.95 ^d^	88.0 ± 2.83 ^c^	88.0 ± 4.24 ^a^

Note: Different letters represent significance in the same column (*p* < 0.05).

**Table 4:** Effect of Fv-SDF addition on the apparent color of dried noodles.

Fv-SDF Addition/%	L*	a*	b*
100.0 (Fv-SDFpowder)	38.21 ± 7.75 ^f^	12.75 ± 0.40 ^a^	27.88 ± 3.92 ^a^
0	88.25 ± 1.47 ^a^	0.19 ± 0.05 ^e^	9.89 ± 0.34 ^d^
5.0	64.32 ± 0.86 ^b^	5.51 ± 0.41 ^d^	13.61 ± 0.78 ^c^
7.5	58.34 ± 1.51 ^c^	6.56 ± 0.78 ^c^	13.74 ± 0.39 ^c^
10.0	52.25 ± 0.65 ^d^	8.92 ± 0.56 ^b^	17.18 ± 0.98 ^b^
12.5	47.80 ± 1.04 ^e^	9.53 ± 0.38 ^b^	17.23 ± 0.48 ^b^
15.0	46.84 ± 0.67 ^e^	9.56 ± 0.22 ^b^	17.52 ± 0.79 ^b^

Note: Different letters represent significance in the same column (*p* < 0.05).

**Table 5:** Analysis of volatile flavor compounds in three samples of dried noodles.

	Name	CAS	Rt [s]	Molecular	Odor Description
Alcohols	1-octene-3-ol	C3391864	867.68	C_8_H_16_O	Mushroom aroma
	1-hexanol-M	C111273	713.00	C_6_H_14_O	Fresh, fruity, alcohol,
	1-hexanol-D	C111273	712.41	C_6_H_14_O	Fresh, fruity, alcohol,
	1-Pentanol-M	C71410	533.20	C_5_H_12_O	balm
	1-Pentanol-D	C71410	532.28	C_5_H_12_O	balm
	1-butanol, 3-methyl-M	C123513	457.43	C_5_H_12_O	Whisky, banana fruit
	1-butanol, 3-methyl-D	C123513	456.99	C_5_H_12_O	Whisky, banana fruit
	1-pentene-3-ol	C616251	388.08	C_5_H_10_O	Ethereal, green, tropical fruit
	1-butanol-M	C71363	369.98	C_4_H_10_O	Red wine taste
	1-butanol-D	C71363	369.04	C_4_H_10_O	Red wine taste
	1-propanol, 2-methyl-M	C78831	311.92	C_4_H_10_O	Fresh, wine, leather
	1-propanol, 2-methyl-D	C78831	310.98	C_4_H_10_O	Fresh, wine, leather
	1-propanol-M	C71238	260.59	C_3_H_8_O	Alcohol, pungent odor
	1-propanol-D	C71238	259.72	C_3_H_8_O	Alcohol, pungent odor
	Ethyl alcohol	C64175	191.40	C_2_H_6_O	toasty
	2-fluoro-uranyl alcohol	C98000	1358.68	C_5_H_6_O_2_	toasty
	1-octanol-M	C124130	609.80	C_8_H_16_O	Aldehyde, waxy, fruity, fat
	1-octanol-D	C124130	609.23	C_8_H_16_O	Aldehyde, waxy, fruity, fat
	2-Pentanol	C6032297	339.50	C_5_H_12_O	Fusel oil, fragrant
	2-butanol	C78922	228.99	C_4_H_10_O	fruity
Aldehyde	Benzaldehyde -M	C100527	957.31	C_7_H_6_O	Bitter almond, cherry and nut notes
	Benzaldehyde D	C100527	957.31	C_7_H_6_O	Bitter almond, cherry and nut notes
	(E)-2-octenal -M	C2548870	809.46	C_8_H_14_O	Fresh cucumber, fat, green fragrance
	(E)-2-octenal-D 1-nonaldehyde-D	C2548870	807.91	C_8_H_14_O	Fresh cucumber, fat, green fragrance
	1-nonanal-M	C124196	759.05	C_9_H_18_O	Rose, citrus, etc
	1-nonaldehyde-D	C124196	759.05	C_9_H_18_O	Rose, citrus, etc
	(E)-2-heptenaldehyde-M	C18829555	660.72	C_7_H_12_O	Green fragrant vegetables, fresh, fat
	(E)-2-heptenaldehyd-D	C18829555	662.94	C_7_H_12_O	Green fragrant vegetables, fresh, fat
	(E)-2-hexene-1-aldehyde-M	C6728263	476.27	C_6_H_10_O	Green fragrant vegetables, fresh, fat
	(E)-2-hexene-1-aldehyde-D	C6728263	476.27	C_6_H_10_O	Green fragrant vegetables, fresh, fat
	3-methyl-2-butenal	C107868	447.79	C_5_H_8_O	fruity
	Heptaldehyde -M	C111717	424.13	C_7_H_14_O	Fresh, fat, green and fruity
	Heptyl aldehyde D	C111717	425.00	C_7_H_14_O	Fresh, aldehyde, fat, green,
	1-hexaldehyde-M	C66251	302.95	C_6_H_12_O	Fresh, green, fat,
	1-hexaldehyde-D	C66251	302.16	C_6_H_12_O	Fresh, green, fat,
	(E)-2-butenal	C123739	267.24	C_4_H_6_O	null
	n-valeraldehyde	C110623	221.23	C_5_H_10_O	Grass smell, taste exciting
	Propanal-M	C123386	134.93	C_3_H_6_O	Pungent smell, grass smell
	Propional-D	C123386	135.47	C_3_H_6_O	Pungent smell, grass smell
	3-methylbutyraldehyde	C590863	182.31	C_5_H_10_O	Chocolate, fat
	(E, E)-2, 4-octanedienal	C30361285	1256.70	C_8_H_12_O	Fat, green, pear, melon
	2-furfural-M	C98011	878.87	C_5_H_4_O_2_	Sweet, wood, toast
	2-furfural-D	C98011	879.98	C_5_H_4_O_2_	Sweetness, wood, bread
Ketone	2-methyl-2-heptene-6-one	C110930	683.56	C_8_H_14_O	Citrus, fruity, keto
	1-hydroxy-2-acetone-M	C116096	628.11	C_3_H_6_O_2_	Pungent smell, caramel taste
	1-hydroxy-2-acetone-D	C116096	628.57	C_3_H_6_O_2_	Pungent smell, caramel taste
	3-hydroxy-2-butanone-M	C513860	599.09	C_4_H_8_O_2_	Buttery, creamy
	2-butanone, 3-hydroxy-D	C513860	599.09	C_4_H_8_O_2_	Buttery, creamy
	2-heptanone	C110430	411.30	C_7_H_14_O	Pear, banana like fruit
	2-butanone-M	C78933	176.41	C_4_H_8_O	Fruity, camphor aromas
	2-butanone-D	C78933	177.48	C_4_H_8_O	Fruity, camphor aromas
	2-acetone	C67641	144.05	C_3_H_6_O	Fresh, apple, pear
Esters	Hexyl propionate	C2445763	671.85	C_9_H_18_O_2_	Sweet fruity smell, soil fragrance
	Butyl acetate	C123864	287.17	C_6_H_12_O_2_	fruity
	Ethyl acetate -M	C141786	169.26	C_4_H_8_O_2_	Fresh, fruity, sweet
	Ethyl acetate -D	C141786	169.08	C_4_H_8_O_2_	Fresh, fruity, sweet

## Data Availability

The original contributions presented in the study are included in the article/Supplementary Material, further inquiries can be directed to the corresponding author.

## Supplementary Materials

The following supporting information can be downloaded at: https://www.mdpi.com/article/10.3390/foods13172764/s1, Table S1. Sensory evaluation.

## References

[1] Yeh M.Y., Ko W.C., Lin L.Y. (2014). Hypolipidemic and antioxidant activity of enoki mushrooms (*Flammulina velutipes*). Biomed. Res. Int..

[2] Liu X.B., Xia E.H., Li M., Cui Y.Y., Wang P.M., Zhang J.X., Xie B.G., Xu J.P., Yan J.J., Li J. (2020). Transcriptome data reveal conserved patterns of fruiting body development and response to heat stress in the mushroom-forming fungus *Flammulina filiformis*. PLoS ONE.

[3] Wei Q., Pan X., Li J., Jia Z., Fang T., Jiang Y. (2021). Isolation and Molecular Identification of the Native Microflora on *Flammulina velutipes* Fruiting Bodies and Modeling the Growth of Dominant Microbiota (*Lactococcus lactis*). Front. Microbiol..

[4] Wang R., Zhang Y., Lu H., Liu J., Song C., Xu Z., Yang H., Shang X., Feng T. (2022). Comparative Aroma Profile Analysis and Development of a Sensory Aroma Lexicon of Seven Different Varieties of *Flammulina velutipes*. Front. Nutr..

[5] Tsai S.-Y., Hwang B.-F., Wang Y.-H., Lin C.-P. (2017). Moisture desorption and thermal properties of polysaccharide from pulsed light irradiated *Flammulina velutipes*. J. Therm. Anal. Calorim..

[6] Liang Q., Zhao Q., Hao X., Wang J., Ma C., Xi X., Kang W. (2022). The Effect of *Flammulina velutipes* Polysaccharide on Immunization Analyzed by Intestinal Flora and Proteomics. Front. Nutr..

[7] Banerjee D.K., Das A.K., Banerjee R., Pateiro M., Nanda P.K., Gadekar Y.P., Biswas S., McClements D.J., Lorenzo J.M. (2020). Application of Enoki Mushroom (*Flammulina velutipes*) Stem Wastes as Functional Ingredients in Goat Meat Nuggets. Foods.

[8] Cao Y., Tian B., Zhang Z., Yang K., Cai M., Hu W., Guo Y., Xia Q., Wu W. (2022). Positive effects of dietary fiber from sweet potato [*Ipomoea batatas* (L.) Lam.] peels by different extraction methods on human fecal microbiota in vitro fermentation. Front. Nutr..

[9] Bai X., He Y., Quan B., Xia T., Zhang X., Wang Y., Zheng Y., Wang M. (2022). Physicochemical properties, structure, and ameliorative effects of insoluble dietary fiber from tea on slow transit constipation. Food Chem. X.

[10] Li S., Hu N., Zhu J., Zheng M., Liu H., Liu J. (2022). Influence of modification methods on physicochemical and structural properties of soluble dietary fiber from corn bran. Food Chem. X.

[11] Thomas M.S., Calle M., Fernandez M.L. (2023). Healthy plant-based diets improve dyslipidemias, insulin resistance, and inflammation in metabolic syndrome. A narrative review. Adv. Nutr..

[12] Coţovanu I., Mironeasa C., Mironeasa S. (2023). Nutritionally Improved Wheat Bread Supplemented with Quinoa Flour of Large, Medium and Small Particle Sizes at Typical Doses. Plants.

[13] Weng J., Chen M., Shi B., Liu D., Weng S., Guo R. (2023). Konjac glucomannan defends against high-fat diet-induced atherosclerosis in rabbits by promoting the PI3K/Akt pathway. Heliyon.

[14] Qi J., Gao J., Zhang Y., Hou W., Han T., Sun C. (2022). The Association of Dietary Fiber Intake in Three Meals with All-Cause and Disease-Specific Mortality among Adults: The U.S. National Health and Nutrition Examination Survey, 2003–2014. Nutrients.

[15] Elleuch M., Bedigian D., Roiseux O., Besbes S., Blecker C., Attia H. (2011). Dietary fibre and fibre-rich by-products of food processing: Characterisation, technological functionality and commercial applications: A review. Food Chem..

[16] Pankiewicz U., Zielińska E., Sobota A., Wirkijowska A. (2022). The Use of Saccharomyces cerevisiae Supplemented with Intracellular Magnesium Ions by Means of Pulsed Electric Field (PEF) in the Process of Bread Production. Foods.

[17] Huber E., Francio D.L., Biasi V., Mezzomo N., Ferreira S.R. (2016). Characterization of vegetable fiber and its use in chicken burger formulation. J. Food Sci. Technol..

[18] Iversen K.N., Jonsson K., Landberg R. (2022). The Effect of Rye-Based Foods on Postprandial Plasma Insulin Concentration: The Rye Factor. Front. Nutr..

[19] Lin Q., Ren A., Liu R., Xing Y., Yu X., Jiang H. (2022). Flavor properties of Chinese noodles processed by dielectric drying. Front. Nutr..

[20] Adamczyk G., Posadzka Z., Witczak T., Witczak M. (2023). Comparison of the Rheological Behavior of Fortified Rye-Wheat Dough with Buckwheat, Beetroot and Flax Fiber Powders and Their Effect on the Final Product. Foods.

[21] Hu Z., Guo W., Liu C., Wang X., Hong J., Liu M., Sun B., Zheng X. (2024). Effect of polysaccharide on rheology of dough, microstructure, physicochemical properties and quality of fermented hollow dried noodles. LWT.

[22] Yan J.K., Wu L.X., Cai W.D., Xiao G.S., Duan Y., Zhang H. (2019). Subcritical water extraction-based methods affect the physicochemical and functional properties of soluble dietary fibers from wheat bran. Food Chem..

[23] Ginindza A., Solomon W.K., Shelembe J.S., Nkambule T.P. (2022). Valorisation of brewer’s spent grain flour (BSGF) through wheat-maize-BSGF composite flour bread: Optimization using D-optimal mixture design. Heliyon.

[24] Guo S., Wu H., Liu X., Zhao W., Zheng J., Li W. (2023). Structural, Physicochemical and Digestive Property Changes of Potato Starch after Continuous and Repeated Dry Heat Modification and Its Comparative Study. Foods.

[25] Ghalambor P., Asadi G., Mohammadi Nafchi A., Seyedin Ardebili S.M. (2022). Investigation of dual modification on physicochemical, morphological, thermal, pasting, and retrogradation characteristics of sago starch. Food Sci. Nutr..

[26] Zhang B., Chen M., Xia B., Lu Z., Khoo K.S., Show P.L., Lu F. (2022). Characterization and Preliminary Application of a Novel Lipoxygenase from *Enterovibrio norvegicus*. Foods.

[27] Huang C., Huang J., Zhang B., Omedi J.O., Chen C., Zhou L., Liang L., Zou Q., Zheng J., Zeng Y. (2023). Rheo-Fermentation Dough Properties, Bread-Making Quality and Aroma Characteristics of Red Bean (*Vigna angularis*) Sourdough Induced by LAB Weissella confusa QS813 Strain Fermentation. Foods.

[28] Kayama K., Wei R., Zhang Y., Wu F., Su Z., Dong J., Liu X. (2022). Effects of Tea Powder on the Cooking Properties, Antioxidative Potential and Volatile Profiles of Dried Noodles. Foods.

[29] Noonim P., Rajasekaran B., Venkatachalam K. (2022). Effect of Palm Oil-Carnauba Wax Oleogel That Processed with Ultrasonication on the Physicochemical Properties of Salted Duck Egg White Fortified Instant Noodles. Gels.

[30] Wang R., Li M., Wei Y., Guo B., Brennan M., Brennan C.S. (2021). Quality Differences between Fresh and Dried Buckwheat Noodles Associated with Water Status and Inner Structure. Foods.

[31] Yeoh S.Y., Tan H.L., Muhammad L., Tan T.C., Murad M., Mat Easa A. (2023). Sensory, structural breakdown, microstructure, salt release properties, and shelf life of salt-coated air-dried yellow alkaline noodles. NPJ Sci. Food.

[32] Hou F., Song S., Cui W., Yu Z., Gong Z., Wang Y., Wang W. (2024). Flavor Improvement of Maillard Reaction Intermediates Derived from Enzymatic Hydrolysates of *Oudemansiella raphanipes* Mushroom. Foods.

[33] Zhou S., Feng D., Zhou Y., Duan H., He Y., Jiang Y., Yan W. (2022). Characteristic Volatile Organic Compound Analysis of Different Cistanches Based on HS-GC-IMS. Molecules.

[34] Guardianelli L.M., Salinas M.V., Brites C., Puppo M.C. (2022). Germination of White and Red Quinoa Seeds: Improvement of Nutritional and Functional Quality of Flours. Foods.

[35] Chipón J., Ramírez K., Morales J., Díaz-Calderón P. (2022). Rheological and Thermal Study about the Gelatinization of Different Starches (Potato, Wheat and Waxy) in Blend with Cellulose Nanocrystals. Polymers.

[36] Wang H., Qiu J., Wu Y., Ouyang J. (2024). Impact of soluble soybean polysaccharide on the gelatinization and retrogradation of corn starches with different amylose content. Food Res. Int..

[37] Zhang L., Chen J., Xu F., Han R., Quan M. (2022). Effect of Tremella fuciformis and Different Hydrocolloids on the Quality Characteristics of Wheat Noodles. Foods.

[38] Liu Y., Wei Z., Wang J., Wu Y., Xu X., Wang B., Abd El-Aty A.M. (2024). Effects of different proportions of erythritol and mannitol on the physicochemical properties of corn starch films prepared via the flow elongation method. Food Chem..

[39] Boonkor P., Sagis L.M.C., Lumdubwong N. (2022). Pasting and Rheological Properties of Starch Paste/Gels in a Sugar-Acid System. Foods.

[40] Li L., Zhou W., Wu A., Qian X., Xie L., Zhou X., Zhang L. (2022). Effect of Ginkgo Biloba Powder on the Physicochemical Properties and Quality Characteristics of Wheat Dough and Fresh Wet Noodles. Foods.

[41] Li S., Chen W., Zongo A.W.S., Chen Y., Liang H., Li J., Li B. (2023). Effects of non-starch polysaccharide on starch gelatinization and digestibility: A review. Food Innov. Adv..

[42] Wang J., He Y., Li X., Xie Y., Wang X., Zhu D., Liu H. (2023). Effect of soluble soybean polysaccharides on the short- and long-term retrogradation properties of instant rice. J. Sci. Food Agric..

[43] Cui C., Caporaso N., Chen J., Fearn T. (2023). Farinograph characteristics of wheat flour predicted by near infrared spectroscopy with an ensemble modelling method. J. Food Eng..

[44] Dufour M., Chaunier L., Lourdin D., Réguerre A.L., Hugon F., Dugué A., Kansou K., Saulnier L., Della Valle G. (2024). Unravelling the relationships between wheat dough extensional properties, gluten network and water distribution. Food Hydrocoll..

[45] Liu M., Chen G., Zhang H., Yu Q., Mei X., Kan J. (2021). Heat-induced inulin-gluten gel: Insights into the influences of inulin molecular weight on the rheological and structural properties of gluten gel to molecular and physicochemical characteristics. Food Hydrocoll..

[46] Liu S., Liu Q., Li X., Obadi M., Jiang S., Li S., Xu B. (2021). Effects of dough resting time on the development of gluten network in different sheeting directions and the textural properties of noodle dough. LWT.

[47] Okami Y., Tsunoda H., Watanabe J., Kataoka Y. (2022). Efficacy of a meal sequence in patients with type 2 diabetes: A systematic review and meta-analysis. BMJ Open Diabetes Res. Care.

[48] Li Q.M., Li Y., Zou J.H., Guo S.Y., Wang F., Yu P., Su X.J. (2020). Influence of Adding Chinese Yam (*Dioscorea opposita* Thunb.) Flour on Dough Rheology, Gluten Structure, Baking Performance, and Antioxidant Properties of Bread. Foods.

[49] Shang J., Zhao B., Liu C., Li L., Hong J., Liu M., Zhang X., Lei Y., Zheng X. (2023). Impact of wheat starch granule size on viscoelastic behaviors of noodle dough sheet and the underlying mechanism. Food Hydrocoll..

[50] Sun H., Zhang Y., Sun J. (2024). Dietary inulin supplementation improves the physicochemical and gel properties of duck myofibrillar protein: Insights into the effect of muscle fiber types. Food Hydrocoll..

[51] Zhang M., Suo W., Deng Y., Jiang L., Qi M., Liu Y., Li L., Wang C., Zheng H., Li H. (2022). Effect of ultrasound-assisted dough fermentation on the quality of dough and steamed bread with 50% sweet potato pulp. Ultrason. Sonochem..

[52] Fan H., Fu F., Chen Y., Liu M., Ai Z., Bian K. (2020). Effect of NaCl on rheological properties of dough and noodle quality. J. Cereal Sci..

[53] Tang P., Zhang S., Meng L., Wang Z., Yang Y., Shen X., Tang X. (2023). Effects of different content of EGCG or caffeic acid addition on the structure, cooking, antioxidant characteristics and in vitro starch digestibility of extruded buckwheat noodles. Int. J. Biol. Macromol..

[54] Zhang X., Wang A., Yao H., Zhou W., Wang M., Liang B., Wang F., Tong L.-T. (2023). Research advancements on the flavor compounds formation mechanism of pickled bamboo shoots in river snails rice noodles. LWT.

