# Supplementary material for: Effect of Flammulina velutipes Soluble Dietary Fiber on Dough Processing Characteristics and Micro-Fermented Dried Noodles Quality Properties
Source: Foods. 2024 Aug 30;13(17):2764. doi: 10.3390/foods13172764 (PMC11395393; doi:10.3390/foods13172764)
Supplement: Supplementary file 1 [file foods-13-02764-s001.zip › foods-3137117-supplementary.pdf]

Table S1. Sensory evaluation

Luster 10	The color of the hanging surface is uniform and glossy (8~10 points);<br>General brightness (6~8 points);<br>Dark color, poor brightness (1~6 points);
Apparent state 10	The surface structure is fine and smooth (8~10 points);<br>The surface structure is finer and smoother (6-8 marks);<br>The surface is rough, swelling, and deformation is serious (1-6 points);
Taste 20	Moderate hardness (17~20 points);<br>Slightly hard or soft (12-17 points);<br>Too hard or too soft (1 to 12 points);
Toughness 15	Has biting strength and elasticity (11~15 points);<br>General bite strength, slightly elastic (5~11 points);<br>Poor bite strength, insufficient elasticity (1~5 points);
Relish 10	Chewing has a strong enoki mushroom and wheat flour flavor (7~10 points);<br>Taste of enokitake mushroom and wheat flour (4 to 7 points);<br>No flammulina flavorful when chewed (1-3 points);
Sticky 15	Refreshing and non-sticky when chewing (11 to 15 minutes);<br>More refreshing, slightly sticky teeth (5~11 points);<br>Not refreshing, sticky (1~5 points);
Palatability 20	Noodles are acceptable and taste good (15~20 points);<br>The acceptance degree of hanging noodles is general, and the taste is general (6~14 points);<br>Do not accept hanging noodles (1-5 points);
